# Analysing First Birth Interval by A CART Survival Tree 

**DOI:** 10.22074/ijfs.2020.6038

**Published:** 2020-10-12

**Authors:** Mahsa Saadati, Arezoo Bagheri

**Affiliations:** National Population Studies and Comprehensive Management Institute, Statistical Methods and Population Modeling Group, Tehran, Iran

**Keywords:** Cox Proportional Hazards Model, First Birth Intervals, Machine learning, Survival Analysis

## Abstract

**Background:**

Birth spacing, especially the first birth interval (FBI), is a suitable index to investigate the delayed fertil- ity that results in a low fertility pattern. Non-parametric familiar alternatives to the Cox proportional hazard regression (CPH) model include survival trees that can automatically discover certain types of covariate interactions according to the survival length. The aim of this research is to study FBI influential factors by applying survival trees.

**Materials and Methods:**

In this cross-sectional study, 610 married women (aged 15-49 years), were selected from different regions of Tehran, Iran in the Winter and Spring of 2017. Classification and regression trees (CART) for the FBI survival tree were fitted by taking into consideration the predictors of each woman’s age, age at first marriage, educational level, partner’s educational level, activity, region, house ownership, kinship, partner’s race, marriage time attitude, and expenditure using R packages.

**Results:**

Since the PH assumption of the CPH model was not confirmed for the covariates of age at first marriage (P=0.001), kinship (P=0.000), partner’s race (P=0.001), and marriage time attitude (P=0.042), the results of this model were not valid. Thus, a CART survival tree was fitted. The validity of the fitted model in assessing FBI was confirmed by the significant result of the log rank test (P<0.01) for the terminal nodes and the value of the separation measure, which was greater than 1. The fitted tree had 13 terminal nodes and the most vital FBI predictor was women’s age. The longest FBI belonged to educated and employed women, ages 30-37 years.

**Conclusion:**

Analysing patterns of birth spacing by selecting the appropriate statistical method provides important informa- tion for health policymakers. In order to formulate appropriate demographic policies, it is essential to take into consideration age, educational level and job status of the women, all of which have essential roles on their decision to have children.

## Introduction

Iran has the lowest fertility rate in the Middle East. In Iran, there has been a reduction in fertility rate from 7 births per woman in 1979 to 1.9 in 2006 and 1.8 in 2011 (1, 2). Delays in childbearing result in a low fertility rate and a decreased fertility pattern in the society. Among a large number of factors that influence the determination of the fertility pattern, it is essential to study the first birth interval (FBI), which is defined as the length of time between two successive live births. FBI is advantageous because the chances for better recall during the post-marriage period or the duration after a woman’s marriage; it is easier for women to remember their first pregnancy information. The delay in the menstrual cycle that occurs after childbearing is not observed in this birth interval. Of note, the other birth intervals are heavily affected by irregular changes in FBI. If women deliver their first child during their younger ages and have shorter ideal birth intervals, it could cause them to have their subsequent children sooner. Thus, these women could most have achieve to their ideal number of children and complete the dimension of their family (3).

Survival analysis comprises a branch of statistical methods that analyse event occurrence and time. Survival analysis has been used to study FBI over the past decade in Iran by using Demographic Health Research (DHS) or survey data. According to the DHS data in 2000, the FBI was 2.7 years (3) and increased to 3.5 years in 2010 (4). Survey data from Semnan Province, Iran in 2012 indicated that the FBI was 2.76 years and 90% of the first children were born four years after the marriage date (5); in Tehran, the FBI increased from 2.5 years in 2000 (2) to 3.2 years in 2017 (6).

Most FBI studies applied non-parametric and semiparametric survival analyses such as Kaplan-Meier (KM) estimations, log-rank tests, and the Cox proportional hazard (CPH) regression model to study factors that impacted FBI. Although simple interpretations of the covariate effects and inferences can be readily achieved by non-parametric survival analysis, they suffer from simultaneously studying the effect of covariates on the response variable. Semi-parametric methods that include CPH and its extensions are used to study survival data and they force a particular connection between the covariates and the response variable. When it is not feasible to define the logarithm of the hazard rate as a linear function of the covariates, more adaptable methods such as survival trees are available. Advantages of survival trees include tremendous flexibility and automatic detection of certain types of covariate interactions without any further specification. Therefore, significant predictive groups of covariates can be easily derived from survival trees. Fitting a single tree has been mostly replaced by an ensemble of trees, which often results in more powerful predictive models that are free from selection of single tree challenges. However, a single tree can still be helpful to gain perception and ease of data interpretation (7).

Many authors have proposed tree-based methods for univariate (or uncorrelated) survival data (8, 9). The development of survival trees has recently grown where the goal was mainly to extend existing tree methods to the case of censored survival data. Classification and regression trees (CART) has gained popularity in many application fields due to the handling of a variety of data structures, the requirement for few statistical assumptions, and ease of interpretation of classification and prediction rules. A CART survival tree has been provided by generalizing the CART algorithm for survival data (8). The main aim of the current research is to study FBI influential factors by applying survival trees as a valid substitution the CPH model when the PH assumption is not fulfilled.

## Materials and Methods

In this cross-sectional study, we investigated factors that influenced women’s FBI so we selected 610 married women, aged 15-49 years from a survey entitled ‘The Effect of Socio-economic Dimensions of Rationality on Childbearing behaviour in Tehran’ in 2017 (10).

The sample size was chosen by the Cochran formula , which took into consideration a 5% error level, proportion of 0.5 (384 samples), a design effect of 2.5 and nonresponse rate of 1.25. Samples were selected by multi-stage stratified random sampling from different regions of Tehran Province, Iran between February and May, 2017. By applying the hierarchical clustering approach, we clustered the regions of Tehran Province according to the developmental indices into four developmental levels: more developed, developed, middle developed, and developing (11). Therefore, the first developmental level consisted of regions 1, 2, 3, and 6, the second developmental level consisted of regions, 5 and 7, the third developmental level consisted of regions 4, 8, 9, 10, 11, 12, 13, 16, 21, 22, 14, and 20, and the forth developmental level consisted of regions 15, 17, 18, and 19. Then, each of the developmental levels in different regions of Tehran was considered as a class; the regions in each of these classes were proportionally selected according to their size. Finally, 10 regions were selected for the final selection. In each selected region, four large blocks were randomly chosen and the samples were collected by systematic random sampling in each block between February and May, 2017. A structured questionnaire that contained demographic, fertility history and childbearing attitudinal factors was completed (10). The validity of the questionnaire was confirmed by 10 demographers and sociologists. Cronbach’s alpha reliability of the questionnaire’s factors was at least 0.771. There were no interventions or treatments in this study, and the aim of the study was explained to the respondents prior to the interview process. The participants provided oral consent to participate in this study and the ethical code was supplied by National Population Studies and Comprehensive Management Institute for the questionnaire (code number: 20/18627). The event of interest was the time of the FBI in months and the main aim of this original study was to detect factors that influenced the women’s FBI.

According to different studies that investigated influential factors on FBI in Iran, the most important covariates included women’s age (6, 12, 13), age at first marriage (1, 14-18), educational level (4-6, 14, 15, 19, 20), partner’s educational level (16, 20), activity (14, 16, 20), region (18, 21), house ownership (16, 19), kinship (21, 22), partner’s race (14, 20), marriage time attitude (18,21), and expenditure (16). According to the literature, we selected the following covariates of women’s age, age at first marriage (<20, 20-29, >30 years), educational level (under diploma, diploma and above), partner’s educational level (under diploma, diploma and above), activity (unemployed, employed), region (developing, middle developing, developed and more developed), house ownership of the family (rent, own, other), kinship (family, non-family), partner’s race (Fars, Turk, other), marriage time attitude (sooner: those who thought that they married sooner than their desired time; later: those thought that they married later than their desired time; and on-time: those who thought that they married at the same desired time), and expenditure (<2 million tomans, 2–3.5 million tomans, ≥3.5 million tomans) were considered. To evaluate the influence of selected covariates on FBI accurately, a CART survival tree method (“rpart” R package) was used for data analysis. With a survival outcome, the splitting criterion used by rpart is equivalent to the one introduced by LeBlanc and Crowley (9).

### Statistical analysis

A CART survival tree can be broadly described as follows:

1. Splitting: Breiman et al. (23) used CART to formalize and generalize the basic idea of recursive binary partitioning of a determined covariate space into smaller regions until a minimum node size could be attained. This is often achieved by minimizing a measure of node impurity. The concluded regions that contain observations of homogeneous response values are called nodes (parent and children nodes). The final partitions are called terminal nodes. For survival data, Ciampi et al. (24) suggested the use of log-rank statistics to compare the two groups formed by the children nodes. The retained split is the one with the largest significant test statistic value. The use of the log-rank test leads to a split which assures the best separation of the median survival times in the two children nodes.

2. Pruning and selection: Backward and forward methods are two approaches used to select a final tree which is not too large to over-fit the data and fail to generalize well to the population of interest, or too small to miss important characteristics of the relationship between the covariates and the outcome. The backward method builds a large tree and then selects an appropriate subtree by pruning and a forward method uses a built-in stopping rule to decide when to stop splitting a node further. The two most important pruning indices are cost-complexity (23) and split-complexity (25).

At the terminal nodes of the selected tree, appropriate node summaries are usually computed to interpret the tree or obtain predicted values. At these nodes, for a categorical outcome, the node proportions of each value will be reported. For a continuous outcome, the node average will be informed and for a survival outcome, the KM estimate of the survival function or the estimated hazard ratios (HR) calculated by the Nelson-Aalen estimator of cumulative hazard function (CHF) (26) will be reported. There is no commonly accepted approach used to assess the predictive ability of the fitted CART survival tree (27). One common approach is to plot the KM estimates for event-free or overall survival in the g groups made by a predictive classification scheme called risk strata or groups. This figure and also significant P-values of the log-rank test when its null hypothesis tests the equality of the survival functions in the g risk strata are necessary, but do not provide sufficient condition for good predictive ability of the fitted CART survival tree. Another approach is to fit a CPH model using dummy variables for the risk strata, and find the estimated HR of the risk strata with respect to a reference group. Crowley et al. (27) proposed a measure of separation (SEP) according to the proportioned absolute estimated logarithm of HR values of the CPH model for the terminal nodes based on a reference node. For survival data, SEP is the standard deviation estimation of the predicted log HR according to a model that has a dummy variable for each group. The favourable tree-based models have greater than 1 value for SEP.

## Results

**Fig.1:**
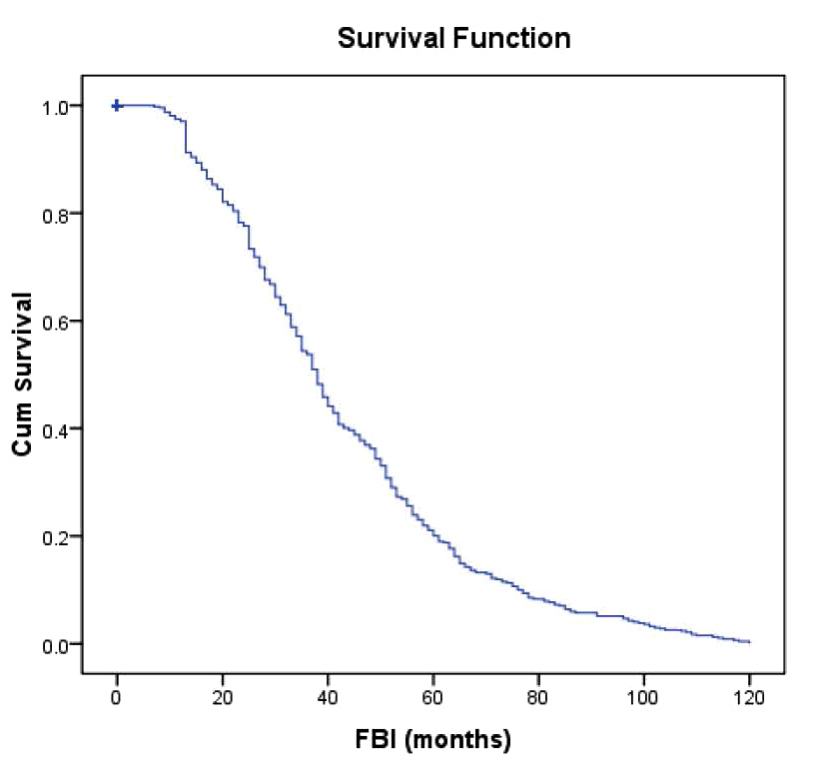
Survival plot of first birth interval (FBI).

In this section, the rationale of applying a CART survival tree in analysing FBI is studied. The dataset includes 469 women with at least one child and 141 censored observations (childless women). It is important to note that the KM mean of the FBI was 38 ± 1.06 months and KM survival estimate plot for the women’s FBI in Figure 1 indicates that most of the women’s the FBI (about 88%) for most women were less than five years.

Table 1 indicates the frequency of women’s demographic statistics and KM estimates. In order to describe the women’s FBI according to the selected covariates in univariate analysis, we used KM estimates and the log-rank test as non-parametric survival tools.

The mean FBI was 42.87 months with a standard deviation of 1.11 months and median of 38 months. The women’s mean age was 35.22 ± 7.91 years and the age at first marriage was 22.61±4.6 years. Table 1 lists the KM means (standard errors) and P value of the log-rank tests for the FBI according to the selected covariates. According to these indicators, it would be easy to define the average and significant differences of this variable amongst the various categories of covariates. Table 1 shows that the women’s age (P<0.001), educational level (P<0.001), partner’s educational level (P=0.001), activity (P=0.014) and region (P=0.020) had significant effects on FBI.

The CPH model was applied to investigate the simultaneous effects of all the covariates on FBI. The PH hypothesis for all covariates was tested by correlating the corresponding set of scaled Schoenfeld residuals with time in order to test for independence between residuals and time. The results are presented in Table 2. A non-significant relationship between residuals and time supports the PH assumption whereas a significant relationship refuses this assumption. Since the PH assumption test of the CPH model was statistically significant for the covariates of age at first marriage (P=0.001), kinship (P<0.001), partner’s race (P=0.001), and marriage time attitude (P=0.042), the PH assumption could not be fulfilled. Therefore, it was unrealistic to expect the reported Cox coefficients to be satisfactory indicators of the actual covariate effects on FBI and the results of the fitted CPH model were not valid.

**Table 1:** Frequency distribution of women’s characteristics and their first birth interval (FBI) Kaplan-Meier (KM) estimates


Variables		Frequency	Percent	KM estimates		
				Median survival time (SD)	Log rank test	P value

Age (Y)						
	<=29	152	24.9	38 (1.72)	22.290	0.000^**^
	30–39	265	43.4	41 (2.47)		
	>=40	193	31.6	34 (1.90)		
Age at first marriage (Y)						
	<20 (ref)	209	34.3	34 (1.58)	4.466	0.107
	20–29	351	57.5	40 (1.35)		
	>30	50	8.2	38 (3.95)		
Educational level						
	Under-diploma	81	13.3	32 (1.59)	13.452	0.000^**^
	Diploma and above	529	86.7	39 (1.10)		
Partner’s educational level						
	Under-diploma	112	18.4	32 (1.65)	10.633	0.001^**^
	Diploma and above	498	81.6	39 (1.12)		
Activity						
	Unemployed	415	68.0	37 (1.22)	6.030	0.014^*^
	Employed	195	32.0	42 (4.13)		
Region						
	Developing and middle developing	419	68.7	37 (1.07)	5.440	0.020^*^
	Developed and more developed	191	31.3	41 (2.83)		
House ownership						
	Renter	307	50.3	38 (1.28)	1.317	0.518
	Owner	238	39.0	37 (1.49)		
	Other	65	10.7	35 (3.82)		
Kinship						
	Family	168	27.5	40 (2.39)	1.328	0.249
	Non-family	442	72.5	37 (1.41)		
Partner’s race						
	Fars	340	55.7	40 (1.40)	5.827	0.054
	Turk	160	26.2	37 (2.25)		
	Other	110	18.0	34 (2.37)		
Marriage time attitude						
	Sooner	63	10.3	33 (2.94)	2.263	0.323
	Later	166	27.2	41 (2.85)		
	On-time	381	62.5	38 (1.12)		
Expenditure						
	<2 million tomans (ref)	362	59.3	37(1.25)	1.765	0.414
	2–3.5 million tomans	176	28.9	41 (2.02)		
	≥3.5 million tomans	72	11.8	36 (1.76)		
Total	610	100.0	38 (1.06)			


*; Significant at the 0.05 level, and **; Significant at the 0.01 level.

**Fig.2:**
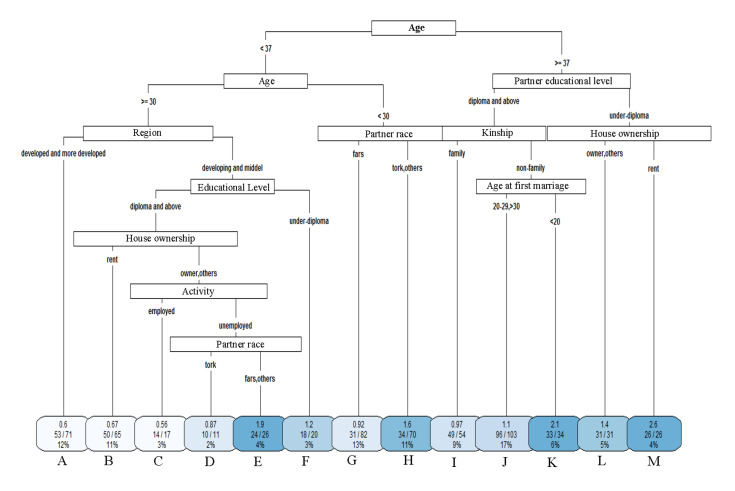
Classification and regression tree (CART) survival tree of first birth interval (FBI). The first line in each terminal node indicates the hazard ratio (HR) within the group. The second line in each node is the number of events and the whole samples on the selected node. The third line are is the percentages of samples in that node. The terminal nodes from left to right are nodes A to M.

We sought to accurately evaluate the influence of selected covariates on FBI by applying a CART survival tree method to the data. The final pruned survival tree selected by cross-validation had 13 terminal nodes and is shown in Figure 2. The first line in each terminal node indicates the HR within the group, the second line in each node is the number of events and the whole samples on the selected node, and the third line is the percentages of samples in that node. The terminal nodes in Figure 2, from left to right, are named nodes A to M. According to the confirmed CART survival tree in Figure 2, the important covariates in analysing FBI were women’s age, partner’s educational level, region, race partner, kinship, house ownership, educational level, age at first marriage, and activity. The first split is based on women’s age. The left node samples are those with age values less than 37 and the right node samples are those with age values greater or equal to 37. Therefore, the terminal nodes that are indicated in Figure 2 by the sorted HR values of FBI are Node C (HR=0.56), Node A (HR=0.6), Node B (HR=0.67), Node D (HR=0.87), Node G (HR=0.92), Node I (HR=0.97), Node J (HR=1.1), Node F (HR=1.2), Node L (HR=1.4), Node H (HR=1.6), Node E (HR=1.9), Node K (HR=2.1), and Node M (HR=2.6). The longest interval between marriage and first birth belongs to the women who were 30-37 years of age and who lived in the developing and middle regions, diploma and above educational level, were owners or had other ownership status, and employed (Node C with an HR=0.56). The shortest interval between marriage and first birth belonged to the 37 years old or older women who had under diploma educational level partner and were renters (node M, with HR=2.6).

**Table 2:** Cox proportional hazard (CPH) model for first birth interval (FBI).


Variables	CPH model			PH assumption test		
	β	Hazard ratio (HR)	Standard error	P value	Chi Square	P value

Age (Y)	0.018	1.019	0.007	0.011^*^	2.443	0.118
Age at first marriage (Y)						
<20 (ref)						
20-29	-0.130	0.878	0.114	0.255	10.747	0.001^**^
>30	0.053	1.054	0.208	0.799	2.166	0.141
Educational level						
Under-diploma (ref)						
Diploma and above	-0.26	0.764	0.170	0.115	1.236	0.266
Partner’s educational level						
Under-diploma (ref)						
Diploma and above	-0.162	0.850	0.143	0.257	0.692	0.405
Activity						
Unemployed (ref)						
Employed	-0.168	0.845	0.119	0.158	0.019	0.889
Region						
Developing and middle developing (ref)						
Developed and more developed	-0.148	0.863	0.110	0.180	1.847	0.174
House ownership						
Renter (ref)						
Owner	0.052	1.054	0.109	0.630	0.007	0.932
Other	0.035	1.035	0.174	0.842	0.251	0.615
Kinship						
Family (ref)						
Non-family	0.318	1.374	0.115	0.005^**^	21.470	0.000^**^
Partner’s race						
Fars (ref)						
Turk	0.241	1.272	0.115	0.037^*^	0.138	0.710
Other	0.118	1.125	0.132	0.373	10.176	0.001^**^
Marriage time attitude						
Sooner (ref)						
Later	-0.206	0.814	0.175	0.240	4.150	0.042^*^
On-time	0.022	1.022	0.162	0.892	0.784	0.376
Expenditure						
<2 million tomans (ref)						
2–3.5 million tomans	-0.005	0.995	0.115	0.964	0.733	0.392
>3.5 million tomans	0.142	1.152	0.176	0.422	0.670	0.413


*; Significant at the 0.05 level and **; Significant at the 0.01 level.

Before further interpretation of the fitted CART survival tree, model validation by sub-group analysis for the terminal nodes was conducted according to two different approaches. Table 3 shows the mean, median and 95% confidence intervals of the FBI along with the log-rank test to compare the FBI in different nodes and significant difference in FBI in the different nodes (P<0.01), which confirmed the validation of the fitted CART survival model according to the first model validation approach.

**Table 3:** Mean, median and log rank test for FBI by nodes


Node	Mean survival time		Median survival time		Log rank test	
	Estimate	95% Confidence interval	Estimate	95% Confidence interval	Chi square	P value

A (HR=0.6)	58.98	(51.03, 66.93)	50.00	(40.83, 59.17)	117.82	0.000^**^
B (HR=0.67)	55.20	(47.81, 62.59)	53.00	(46.07, 59.93)		
C (HR=0.56)	63.21	(45.14, 81.29)	48.00	(0.00, 110.34)		
D (HR=0.87)	47.80	(31.10, 64.50)	33.00	(12.86, 53.14)		
E (HR=1.9)	30.17	(24.15, 36.18)	26.00	(22.40, 29.60)		
F (HR=1.2)	38.33	(28.82, 47.85)	37.00	(26.61, 47.39)		
G (HR=0.92)	46.29	(39.10, 53.48)	48.00	(32.73, 63.27)		
H (HR=1.6)	33.82	(29.10, 38.54)	34.00	(26.86, 41.14)		
I (HR=0.97)	44.65	(39.03, 50.27)	42.00	(33.77, 50.23)		
J (HR=1.1)	41.33	(36.94, 45.73)	38.00	(34.16, 41.84)		
K (HR=2.1)	27.61	(21.81, 33.41)	25.00	(17.79, 32.21)		
L (HR=1.4)	36.68	(31.08, 42.28)	34.00	(30.90, 37.10)		
M (HR=2.6)	25.11	(20.48, 29.75)	23.00	(21.77, 24.23)		
Overall	42.87	(40.69,45.05)	38.00	(35.91, 40.09)		


*; Significant at the 0.05 level, **; Significant at the 0.01 level, and FBI; First birth interval.

**Table 4:** Cox proportional hazard (CPH) model first birth interval (FBI) according to the terminal nodes


Variable Node	CPH model				PH assumption test	
	β	Hazard ratio (HR)	Standard error	P value	Chi square	P value

A	-1.793	0.251	0.251	0.000	0.403	0.525
B	-1.622	0.249	0.249	0.000	0.021	0.885
C	-1.895	0.341	0.341	0.000	0.077	0.781
D	-1.353	0.376	0.376	0.000	0.105	0.745
E	-0.390	0.284	0.284	0.170	0.028	0.866
F	-0.910	0.309	0.309	0.003	1.177	0.278
G	-1.230	0.270	0.270	0.000	1.035	0.309
H	-0.591	0.262	0.262	0.024	1.049	0.306
I	-1.166	0.246	0.246	0.000	0.045	0.832
J	-1.065	0.225	0.225	0.000	1.534	0.215
K	-0.317	0.263	0.263	0.228	0.420	0.517
L	-0.758	0.268	0.268	0.005	0.060	0.806


According to Figure 2, after the first split on women’s age (<37 and ≥37 years), it was clear that the women whose ages ≥37 (especially in nodes J to M) tended to have lower survival time of childlessness and greater hazard rate (>1.1), which resulted in a shorter FBI. The smallest survival time node (node M) was formed by renter women aged ≥37 years with a less than diploma educational level (HR=2.6). Again, after the second split on women’s age, the subjects with a value of between 30 and 37 in nodes A to D tended to have greater survival time of childlessness and lower HR (<0.87), which resulted in longer FBI. The largest survival time node (node C) was formed by women aged 30-37 years who resided in developing and middle regions, had a diploma and above educational level, were owners or had other house ownership status, and were employed (HR=0.56).

In order to study the second model validation approach for the fitted CART survival tree, we computed the SEP measure, which confirms the predictive ability of the fitted model. The CPH model was fitted to the FBI according to the terminal nodes of the resultant tree [Table 4, (27)].

The values of -2log likelihood (4755.845) and P<0.01 of the fitted CPH model indicated the significance of this model. Moreover, the PH assumption test was not statistically significant for all of the terminal nodes, which confirmed the validity of the CPH model (P>0.05). Thus, the resultant coefficient estimations of the CPH model in Table 4 could be valid for computation of the SEP value of the fitted CART survival tree. In order to calculate the SEP value, first, from the second line of terminal nodes in Figure 2, we took into consideration we took into consideration the fractions of the number of risk exposure samples on that node to the whole sample size (this fraction for node A is 71/610 = 0.12). Then, each fraction was multiplied by the coefficients (β) of the CPH model for the terminal nodes in Table 4 and summed. The SEP value could be calculated by computing the exponential of the resultant value. In this study, this value is equal to 2.94 and it is >1, which resulted in the validity of this model (28).

## Discussion

By recently decreasing the total fertility rate (TFR) under the replacement level recently in Iran, many researchers investigated the effect of factors on TFR. One of its most influential factors was birth interval (1-3). The median trend of FBI in Tehran from 2009 (23 months) (2) to 38 months (2017) (6) indicated an ascendant, which demonstrated the need for more researches in this field. Most studies on FBI were conducted by applying the CPH model (5, 14). CPH is a semi-parametric, popular technique for analysing survival data. If the PH assumption (which means the logarithm of the hazard rate is a linear function of the covariates) does not fulfil all the covariates in the real data sets, it is unrealistic to expect the reported Cox coefficients to be satisfactory indicators of the actual covariates. Tree-based or recursive partitioning methods, such as survival trees, are popular non-parametric alternatives to the CPH model they need fewer assumptions, have greater flexibility, are easy to understand, can be explained easily, and inevitably they identify different kinds of covariate interactions. Moreover, based on the covariates, they can cluster subjects according to their length of survival patterns (29-31). Survival trees are a very active ongoing area of research (7).

To the best of our knowledge, no studies have considered influential FBI factors by the CART survival tree. The main aim of this paper was to apply the CART survival tree, to analyse the FBI of 610 married Iranian women, as an alternative non-parametric method for situations where the PH assumption of the CPH model was not fulfilled. According to the results, the KM estimator of the FBI was 38±1.06 months; almost 88% of the women delivered their first child more or less over a five-year interval. Although based on the log-rank test, the women’s age, educational level, their husband’s educational level, region and activity significantly affected their FBI (P<0.05). The results of the fitted CPH model were not reliable due to the unsatisfactory results of the PH assumption test for some of the covariates. In order to consider the simultaneous effects of all covariates on FBI, a CART survival tree was fitted to the data. The validity of the model was confirmed according to the results of the log-rank test (P<0.05) and SEP measure (SEP>1) for terminal nodes of the fitted CART survival tree.

According to the resultant CART survival tree, the root or most influential factor on FBI was the women’s age. In some of the studies, increasing age was a contributing factor to women's fertility, which caused an increase in the incidence of problems and diseases during pregnancy and childbearing played a crucial role in the women's fertility (6, 12, 13). Keshavarz et al. (13) assessed 20.49-year-old married women in Isfahan and noted a reverse correlation with the women’s age and delay in their childbearing, which was in line with the results of this study. The HR of FBI for women aged ≥37 years of age was almost larger than for the women <37 years of age. Thus, the interval between marriage and childbearing for women decreased by increasing their age.

Another important issue is women’s education. Women’s views on marriage and fertility can be influenced by education, in particular the longer duration of university studies. Instead of childbearing, it seems that university educated women concentrated on alternative social roles. The probability of tendency, identifying, and entering women into a range of social activities and technical skills could be increased by education (5). On the other hand, educated women have access to information about how to delay their childbearing and are more likely to be engaged in occupations that are not readily compatible with having children (19). The findings of previous studies in other countries (32, 33), and particularly Iran (4), indicated that increasing women’s educational level resulted in increased FBI. An assessment of the DHS data from 38 out of 51 countries found that illiterate women were more expected to consider a shorter space between their marriage and childbearing compared with educated women (34). A survey conducted in seven Asian countries indicated a negative relationship between women’s educational level and their FBI (33). Iranian studies in Hamedan Province (35), Shiraz Province (15), Ahwaz Province (20), and Tehran Province (6) showed that women’s educational levels were one of the important covariates that had a significant effect on FBI. These results were in line with the results of this study. The FBI for women with a ‘diploma and above’ was almost longer than the under diploma’ educational level (5, 14, 15, 19).

The partner’s educational level had a significant influence on FBI in this study. Most women with educated partners had longer FBI compared to those whose partners were uneducated. Charmzadeh et al. (20) and Alam reported the same result (16).

The results of some studies indicated that age at first marriage was an important and main determinant of FBI (14). In theory, the marriage age is inversely related to FBI, and women who married at a younger age were more likely to have their first birth later (17). Abbasi- Shavazi et al.(1), in a study in Iran, reported that the delay in marriage for women was not desirable, but delayed motherhood due to contraceptive use after marriage was attributed to achieving their goals (1). Some studies indicated that a higher age at first marriage was associated with a decreased risk of long FBI (14, 18, 22, 27, 33). In the current study, the first marriage age variable in the presence of other covariates, partner’s educational level, kinship, and house ownership, in the multivariate analysis influenced FBI but contradicted the above mentioned studies. The covariate of age at first marriage in the fitted CART survival tree was located under the cluster of women aged ≥37 years and was probably due to the interaction effect of partner’s educational level, which resulted in a shorter FBI for women who married younger in this cluster. The same results were also obtained by Erfani and McQuillan (18).

The findings of the current study showed that employed women had children later. This finding supported economic theories. Based on the contradiction between childbearing and economic activity, due to barriers of work and childbearing conflicts, and opportunity costs of childbearing, women's employment would be expected to lead to an increased delay in childbearing and decrease in the number of children. Erfani et al. (14), Charmzadeh et al. (20), and Alam (16) reached the same conclusions.

Region was also a significant factor for FBI in this study. The influential effect of this covariate has been studied in fertility researches (18, 21). Erfani and McQuillan (18) concluded that woman who lived in developing regions compared to more developed regions had shorter FBI. Their findings supported the results of our study.

Another influential factor on FBI was partner’s race. Other studies like Charmzadeh et al. (20) and Erfani et al. (14) evaluated the effect of race on FBI. However, Erfani et al. (14) reported that this covariate was not significant. Charmzadeh et al. (20) concluded that women whose partners were of the Fars race had longer FBI compared to other women. This result was along the same line as this study for women aged <30. However, for women aged 30 to <37 years of age, the women whose partners were of the Fars race had shorter FBI compared to the other women.

House ownership was a significant covariate for FBI in this study. Charmzadeh et al. (20) indicated that renter women had shorter FBI. Alam (16) and Yohannes et al. (36) also studied women’s socio-economic status and its impact on FBI; they concluded that rich women had longer FBI. These studies supported our results in the cluster of women aged ≥37 years. However, in the cluster of women aged <37 years, under the effect of the educational level covariate, we reached a different conclusion. Educated renter women had an almost longer FBI compared to other educated women.

Another significant covariate on FBI was kinship. The influence of this covariate on women’s fertility was studied by Saadati and Bagheri (21) and Bagheri et al. (22). Saadati and Bagheri (21) determined that this covariate did not significantly impact FBI. According to their result, women with family partners had longer FBI compared to women with non-family partners (21).

## Conclusion

The delay in childbearing or increased birth intervals, particularly the first childbearing or FBI, are among the main factors that decreased the fertility rat to low levels. Therefore, it is essential to study the factors that affect FBI. Based on the findings of this study, a reduction in the interval between marriage age and childbearing will not be attained unless policy makers and governors provide appropriate socio-economic conditions for the families, especially in terms of the women ‘s employment and education.

## References

[1] Abbasi-Shavazi MJ, McDonald P, Hosseini-Chavoshi M (2009). The fertility transition in Iran: Revolution and reproduction.

[2] Erfani A (2014). Fertility survey in Tehran, study of TFR changes attitudes and preferences of childbearing.

[3] Abbasi-Shavazi M, Razeghi-Nasrabad HB (2012). Patterns and factors affecting marriage to first birth interval in Iran. Journal of Population Association of Iran.

[4] McDonald P, Hosseini-Chavoshi M, Abbasi-Shavazi MJ, Rashidian A (2015). An assessment of recent Iranian fertility trends using parity progression ratios. Dem Res.

[5] Saadati M, Bagheri A (2015). Razeghi-Nasrabad H.The first birth interval and its determinants in Semnan province by the parametric survival Analysis model. Journal of Population Association of Iran.

[6] Saadati M, Bagheri A, Abdolahi A (2018). Marriage to first birth interval; A Cross-sectional study in Tehran (Iran). IJWHR.

[7] Bou-Hamad I, Larocque D, Ben-Ameur H (2011). A review of survival trees. Statist Surv.

[8] Gordon L, Olshen RA (1985). Tree-structured survival analysis. Cancer Treat Rep.

[9] LeBlanc M (1992). Crowley J.Relative risk trees for censored survival data. Biometrics.

[10] Abdolahi A (2017). Effects of socio-economic rationality dimensions on childbearing behaviour in Tehran.

[11] Rafieian MS (2012). The spatial analysis of Tehran’s development level based on metropolitan areas. J Spat Plan.

[12] Amerian M, Kariman NS, Janati P, Salmani F (2016). The role of individual factors in deciding the first childbirth. Payesh.

[13] Keshavarz H, Haghighatiyan M, Tavasoli Dinani KH (2013). Investigating the factors affecting the interval between marriage and childbearing (case study: 20-49-year-old married women in Isfahan). Apple Social.

[14] Erfani A, Nojomi M, Hosseini H (2018). Prolonged birth intervals in Hamedan, Iran: variations and determinants. J Biosoc Sci.

[15] Shayan Z, Ayatollahi SMT, Zare N, Moradi F (2014). Prognostic factors of first birth interval using the parametric survival models. Iran J Reprod Med.

[16] Alam MM (2015). Marriage to first birth interval and its associated factors in Bangladesh. AJSSH.

[17] Soltanian A (2019). Davar S.Akhgar MM.Mahjub H.Karami M.Modeling the factors affecting the first birth in the family's fertility in Hamedan province. J Pharm Res Int.

[18] Erfani A, McQuillan K (2014). The changing timing of births in Iran: an explanation of the rise and fall in fertility after the 1979 Islamic Revolution. Biodemography Soc Biol.

[19] Begna Z, Assegid S, Kassahun W, Gerbaba M (2013). Determinants of inter birth interval among married women living in rural pastoral communities of southern Ethiopia: a case control study. BMC Pregnancy Childbirth.

[20] Charmzadeh R, Akhond MR, Rasekh AR (2014). Factors affecting the birth intervals: the case of women referred to health centres in Ahwaz province. Hayat.

[21] Saadati M, Bagheri A (2018). Analysing birth interval by recurrent event models.

[22] Bagheri A, Saadati M, Razeghi Nasrabad HBB (2014). Introduction and application of CART tree algorithm to classify Ideal number of children of 15-49 old-year women in Semnan province. Popul Assoc Lett.

[23] Breiman L, Friedman J (1984). Olshen R, Stone C.Classification and regression trees.New York: Chapman and Hall, Wadsworth.

[24] Ciampi A, Thiffault J, Nakache JP (1986). Asselain, B.Stratification by stepwise regression, correspondance analysis and recursive partition: a comparison of three methods of analysis for survival data with covariates. Comput Stat Data An.

[25] Leblanc M, Crowley J (1993). Survival trees by goodness of split. J Am Stat Assoc.

[26] Ishwaran H, Kogalur UB (2010). Consistency of random survival forests. Stat Probab Lett.

[27] Crowley J, Hoering A, Ankerst D (2005). Handbook of statistics in clinical oncology.

[28] Saki Malehi A, Hajizadeh E, Fatemi R (2012). Evaluation of prognostic variables for classifying the survival in colorectal Patients using the decision tree. Iran J Epidemiology.

[29] Zhang H, Singer B (2010). Recursive partitioning and applications.Berlin: Springer Science and Business Media.

[30] Parizadeh D, Ramezankhani A, Momenan AA, Azizi F, Hadaegh F (2017). Exploring risk patterns for incident ischemic stroke during more than a decade of follow-up: a survival tree analysis. Comput Methods Programs Biomed.

[31] Ramezankhani A, Bagherzadeh-Khiabani F, Khalili D, Azizi F, Hadaegh F (2017). A new look at risk patterns related to coronary heart disease incidence using survival tree analysis: 12 years longitudinal study. Sci Rep.

[32] Setty-Venugopal V, Upadhyay UD (2002). Birth spacing.Three to five saves lives. Popul Rep L.

[33] MacQuarrie K (2017). Trends and factors associated with marriage timing and the first birth interval.Chicago; Population Association of America Annual Meetings.

[34] Shimokawa A, Kawasaki Y, Miyaoka E (2015). Comparison of splitting methods on survival tree. Int J Biostat.

[35] Najafi-Vosough R, Soltanian AR, Fayyazi N (2017). Influence factors on birth spacing and childbearing rates using survival recurrent events model and parity progression ratios. J Res Health Sci.

[36] Yohannes S, Wondafrash M, Abera M, Girma E (2011). Duration and determinants of birth interval among women of child bearing age in Southern Ethiopia. BMC Pregnancy Childbirth.

